# *Aedes aegypti* breeding ecology in Guerrero: cross-sectional study of mosquito breeding sites from the baseline for the Camino Verde trial in Mexico

**DOI:** 10.1186/s12889-017-4293-9

**Published:** 2017-05-30

**Authors:** Arcadio Morales-Pérez, Elizabeth Nava-Aguilera, Alejandro Balanzar-Martínez, Antonio Juan Cortés-Guzmán, David Gasga-Salinas, Irma Esther Rodríguez-Ramos, Alba Meneses-Rentería, Sergio Paredes-Solís, José Legorreta-Soberanis, Felipe Gil Armendariz-Valle, Robert J. Ledogar, Anne Cockcroft, Neil Andersson

**Affiliations:** 1 Centro de Investigación de Enfermedades Tropicales (CIET), Universidad Autónoma de Guerrero, Acapulco, Guerrero Mexico; 2 Departamento de Prevención y Control de Enfermedades Transmisibles por Vector, Servicios Estatales de Salud Guerrero, Av. Rufo Figueroa 6, Colonia Burócratas, Chilpancingo, Guerrero Mexico; 3 CIETinternational, New York, NY USA; 4 Department of Family Medicine, McGill University, Montreal, Canada; 5 CIET Trust, Gaborone, Botswana

**Keywords:** *Aedes aegypti*, Dengue, Pupal productivity, Temephos

## Abstract

**Background:**

Understanding the breeding patterns of *Aedes aegypti* in households and the factors associated with infestation are important for implementing vector control. The baseline survey of a cluster randomised controlled trial of community mobilisation for dengue prevention in Mexico and Nicaragua collected information about the containers that are the main breeding sites, identified possible actions to reduce breeding, and examined factors associated with household infestation. This paper describes findings from the Mexican arm of the baseline survey.

**Methods:**

In 2010 field teams conducted household surveys and entomological inspections in 11,995 households from 90 representative communities in the three coastal regions of Guerrero State, Mexico. We characterized *Ae. aegypti* breeding sites and examined the effect of two preventive measures: temephos application in water containers, and keeping the containers covered. We examined associations with household infestation, using bivariate and multivariate analysis adjusted for clustering effects.

**Results:**

We conducted entomological inspections in 11,995 households. Among 45,353 water containers examined, 6.5% (2958/45,353) were positive for larvae and/or pupae. Concrete tanks (*pilas*) and barrels (*tambos*) together accounted for 74% of pupal productivity. Both covering water containers and inserting temephos were independently associated with a lower risk of presence of larvae or pupae, with the effect of covering (OR 0.22; 95% CIca 0.15–0.27) stronger than that of temephos (OR 0.66; 95% CIca 0.53–0.84). Having more than four water containers was associated with household infestation in both rural areas (OR 1.42; 95% CIca 1.17–1.72) and urban areas (1.81; 1.47–2.25), as was low education of the household head (rural: 1.27; 1.11–1.46, and urban: 1.39; 1.17–1.66). Additional factors in rural areas were: household head without paid work (1.31; 1.08–1.59); being in the Acapulco region (1.91; 1.06–3.44); and using anti-mosquito products (1.27; 1.09–1.47). In urban areas only, presence of temephos was associated with a lower risk of household infestation (0.44; 0.32–0.60).

**Conclusion:**

Concrete tanks and barrels accounted for the majority of pupal productivity. Covering water containers could be an effective means of *Ae. aegypti* vector control, with a bigger effect than using temephos. These findings were useful in planning and implementing the Camino Verde trial intervention in Mexico.

## Background

Dengue is an important neglected infectious disease in the Latin America and Caribbean region [1]. Globally, there are an estimated 390 million dengue infections a year, of which 96 million show clinical manifestations ranging from mild to the most severe [2]. The main dengue vector is the *Aedes aegypti* mosquito, which is drawn to urban habitats and reproduces mainly in artificial water containers inside or outside households [3]. Irregular or low-quality household water supplies compel households to store water in tanks, barrels or other containers, often open, thus creating a favourable environment for the female *Ae. aegypti* to lay their eggs and turn them into sites for their offspring to develop [4, 5]. Low quality urban development contributes to the proliferation of containers in which *Ae. aegypti* breed [6, 7].

Many types of container can become breeding sites [8], and their contribution to mosquito production may vary depending on the season [9, 10]. Certain household characteristics have been identified as adding to the risk of infestation by immature forms of *Ae. aegypti*, such as the number of people living in a household and the household head’s educational level [11] and gender [12].

This article reports an analysis of data from the baseline survey for Camino Verde, a cluster-randomised controlled trial to reduce dengue risk in southern Mexico and Nicaragua through evidence-based community mobilisation, conducted in 2010 and described by Andersson et al. [13]. Other community mobilisation interventions have shown impact on entomological indicators; Camino Verde was the first to have demonstrated impact against dengue virus infection and reported cases of dengue illness. The baseline for the Mexican arm of the trial collected information from households in 90 clusters, representative of the three coastal regions of Guerrero State. Findings from the baseline regarding the *Ae. aegypti* breeding sites in and around households, and the factors related to infestation, were an important contribution to the evidence-based community mobilisation intervention in the trial. This article describes the types of water containers found in the households, documents the levels of infestation with *Ae.aegypti* larvae and pupae, and examines the factors associated with infestation.

## Methods

The baseline study for the Camino Verde trial [13] covered the three coastal regions in State of Guerrero: Acapulco, Costa Grande and Costa Chica. The regions have a warm and sub-humid climate with a mean yearly temperature of 25 °C. Total annual precipitation averages 1387 mm, concentrated mostly in the rainy season from June to September. The Costa Grande has a population of 384,534, Costa Chica 449,360, and Acapulco 789,971. These three regions make up 48% of the State’s population [14].

The methods of the baseline study for the trial are described in detail elsewhere [13, 15]. There were three elements: a community profile, based on observation and interviews with key informants, which documented relevant characteristics of each community; a household survey administered to a respondent in each household (an average of 137 households per cluster); and an entomological survey of each household.

### Entomological survey

At the time of the household survey, which was conducted during the dry season of 2010–2011, trained entomological fieldworkers, accompanied by a household member, examined all water containers on the property. These workers had at least a high school education and most had a first university degree (*licenciatura*); nearly all had experience working for the vector control programme of the Guerrero state health department. They all underwent a special 20-h course on the biology of the mosquito and how to search for mosquito reproduction sites, delivered by experienced medical entomologists. Their field supervisors also had entomological experience.

The fieldworkers extracted every larva and pupa they found in the containers and transported them in labelled plastic bags, in thermos flasks, to the laboratory at the *Centro de Investigación de Enfermedades Tropicales* (CIET) in the University of Guerrero. At the laboratory, entomologists stored the bags at −20 °C, counted the larvae and pupae and classified them using stereoscopic microscopes. They recorded the total numbers of *Ae. aegypti* larvae and pupae for each container. We considered water containers positive for *Ae. aegypti* infestation if they contained at least one larva or pupa. Similarly, we considered households infested when we found at least one larva or pupa in at least one container on the property.

The entomological inspections also provided information on the number of containers, their locations, if they were covered or uncovered, the household use of the water in each container, presence of the chemical larvicide, *temephos*, and the time since the temephos application. Whenever inspectors identified temephos in a container they asked the member of the household accompanying them how long ago it was inserted.

### Data management and analysis

Data entry relied on Epi-Data 3.1 [16] open-source software, with double data entry and validation to minimize keystroke errors. We conducted the data analysis using the CIETmap open source software package [17, 18], which provides a user-friendly interface with the R statistical programming language.

We calculated several entomological indices: the container index (the number of positive containers divided by the total number of containers examined); the household index (the number of households with at least one positive container divided by the total number of households); the Breteau index (the number of positive containers divided by the total number of households); and the pupa per person index (the number of pupae in a defined area divided by the population in that area).

We examined the association between two preventive actions - placing temephos in containers and covering containers – and the presence of any *Ae. aegypti* larvae or pupae in the container, taking account of both actions together using the Mantel Haenszel procedure [19], and adjusting for clustering using the method of Lamothe [20]. We considered a container to have “active temephos” when the larvicide had been placed in the container less than 2 months previously.

A bivariate analysis examined factors potentially associated with household infestation by immature forms of *Ae. aegypti*, with household index as the outcome variable. The factors we examined included: region of residence and whether urban or rural, type of housing and its use, language spoken at home, regularity of water supply and refuse collection, number of receptacles containing water in the household, presence of temephos in any of the containers, household use of anti-mosquito products, employment status of the household head, education of the household head, and respondent knowledge of the dengue vector. We then carried out a multivariate analysis, using the Mantel-Haenszel procedure [19] with cluster adjustment [20]. The initial saturated model included all variables associated with the outcome in bivariate analysis, and we employed step-wise deletion of the least significant association to reach a final model in which all the variables were significantly associated with the outcome at the 5% level. We tested for effect modification with the Woolf χ^2^ test for heterogeneity [21]. There was significant effect modification by urban/rural status and we therefore created separate multivariate models for urban and rural areas. We express associations using the adjusted odds ratio (OR) and the cluster adjusted 95% confidence interval (95% CIca).

## Results

**Fig. 1:**
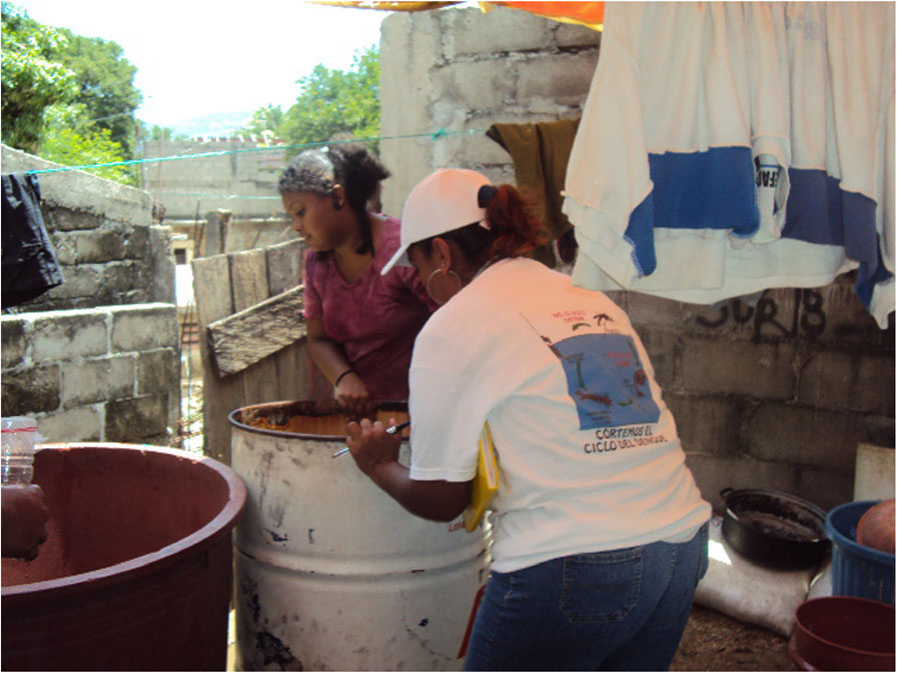
Two barrels (*tambos*) for water storage, one plastic and one metal

**Fig. 2:**
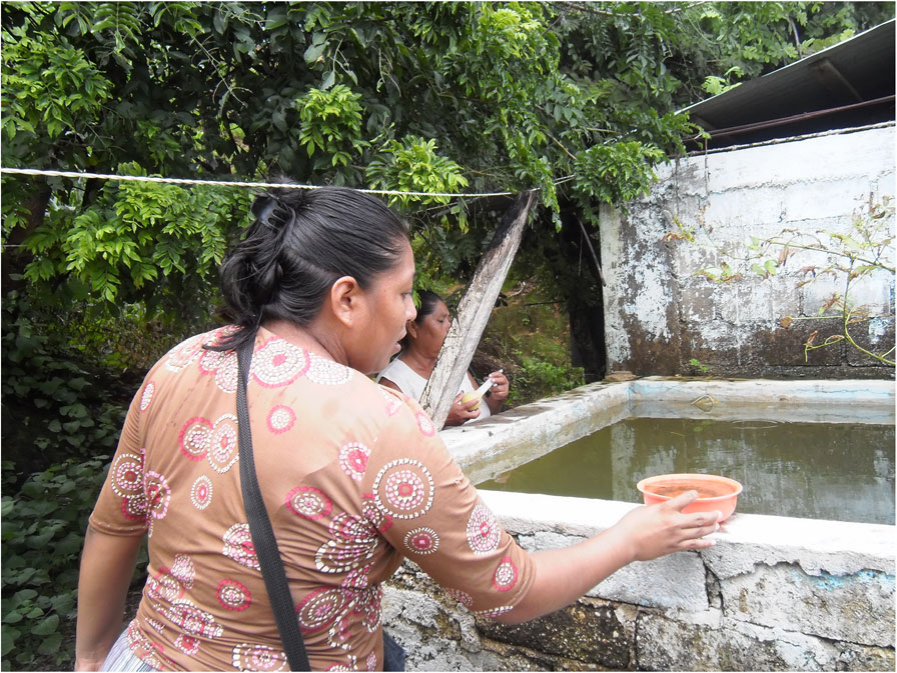
An uncovered concrete water storage tank (*pila*)

**Fig. 3:**
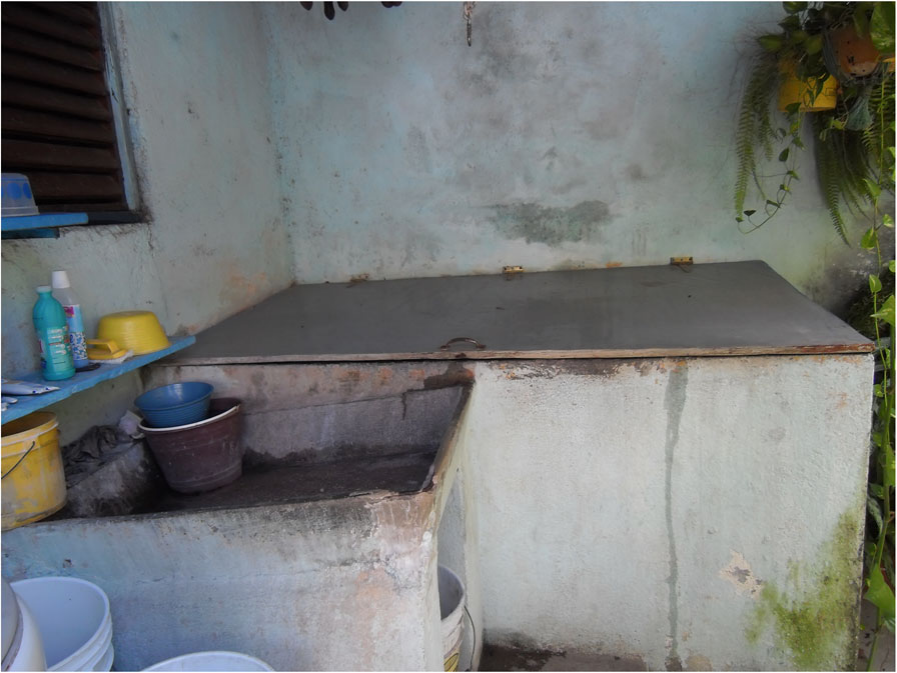
A covered concrete water storage tank (*pila*)

**Fig. 4:**
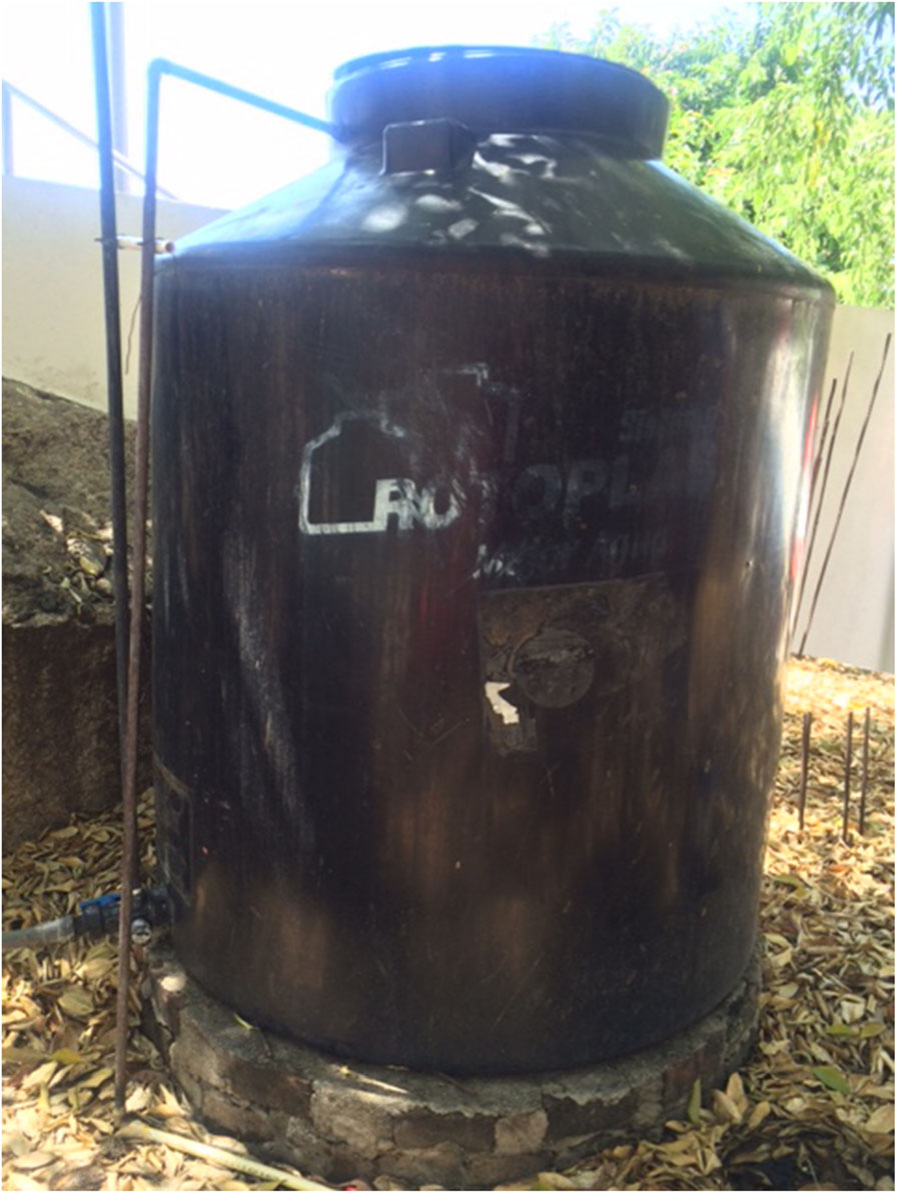
A plastic water tank (*tinaco*)

**Fig. 5:**
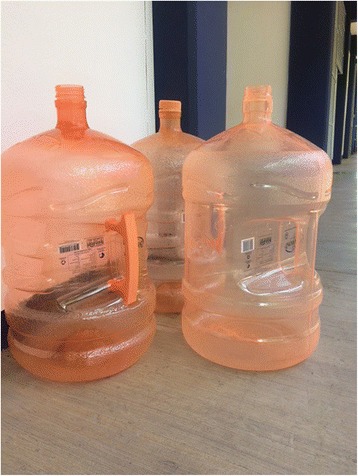
Plastic bottles for drinking water (*garrafones*)

The field teams conducted an entomological inspection in 11,995 households (97% of the households who participated in the household interviews) and found 45,353 containers which held water at the time. These included containers used for water storage, as well as some receptacles not used for water storage but in which water had accumulated. Overall, the most common were barrels/drums (*tambos*) (36.9%) (Figure 1), concrete tanks (*pilas)* (20.6%) (Figs. 2 and 3) and buckets (*cubetas*) (19.2%) (Table 1). Other containers included large plastic tanks (*tinacos*) (Figure 4) and plastic bottles (*garrafones)* (Figure 5). There was some variation in the pattern of containers across the three regions, as shown in Table 1.

**Table 1:** Different types of water containers examined in the three regions

Container type	Number (%) of containers			
	Acapulco	Costa Grande	Costa Chica	All regions
Water storage containers				
Barrels^a^	6379 (36.5)	4984 (37.3)	5352 (36.9)	16,715 (36.9)
Concrete tanks^b^	3021 (17.3)	3398 (25.2)	2939 (20.3)	9358 (20.6)
Buckets^c^	4688 (26.8)	1873 (14.0)	2159 (14.9)	8720 (19.2)
Other containers^d^	863 (4.9)	1116 (8.4)	2622 (18.1)	4601 (10.1)
Plastic tanks^e^	1684 (9.6)	1176 (8.8)	509 (3.5)	3369 (7.4)
Plastic bottles^f^	492 (2.8)	728 (5.5)	335 (2.3)	1555 (3.4)
Containers not used for water storage				
Discarded articles^g^	236 (1.3)	48 (0.4)	334 (2.3)	618 (1.4)
Tyres	25 (0.1)	5 (0.04)	183 (1.3)	213 (0.5)
Plant and flowerpots	106 (0.6)	21 (0.2)	77 (0.5)	204 (0.4)
Total	17,494 (100)	13,349 (100)	14,510 (100)	45,353 (100)

Terminology for water containers varies, even between states in Mexico. The terminology in this article is that used in Guerrero State

^a^Barrels or drums (*tambos*) are made of plastic or metal and hold about 2000 l (Figure 1)

^b^Concrete/cement tanks (*pilas*) are of variable size, up to thousands of litres (Figs. 2 and 3)

^c^Buckets (*cubetas*) hold between 20 l and 200 l

^d^Other containers used for water storage include washtubs, trays, and various kitchen utensils

^e^Large plastic tanks (*tinacos*) are made of heavy plastic with capacity 450 l to 10,000 l (Figure 4)

^f^Plastic bottles (*garrafones*) – usually hold between 5 l and 20 l (Figure 5)

^g^Discarded articles (*cacharros*) with configuration allowing water accumulation

Overall, 31% of containers were covered, with little difference between urban and rural areas (Table 2). While most concrete tanks are found outside the household’s immediate living space, most have some kind of roof or overhang to protect them from the rain. Nevertheless, only 8% of concrete tanks were covered --12% in urban areas and just 4% in rural areas. About a third of barrels and a quarter of buckets were covered. Most plastic tanks (*tinacos*) and water bottles were covered or sealed in both rural and urban areas.

**Table 2:** Distribution of water storage containers and presence of covers, in urban and rural areas

Type of container	% (proportion) of containers that were covered		
	Urban sites	Rural sites	Total
Barrels/drums	31 (2034/6499)	37 (3659/9783)	35 (5693/16282)
Concrete tanks	12 (573/4620)	4 (183/4571)	8 (756/9191)
Buckets	27 (1177/4426)	23 (963/4151)	25 (2140/8577)
Plastic tanks	87 (1805/2070)	82 (998/1224)	85 (2803/3294)
Plastic bottles	81 (691/852)	82 (542/662)	81 (1233/1514)
Other storage containers	7 (92/1229)	17 (543/3270)	14 (635/4499)
All storage containers	32 (6372/19696)	29 (6888/23661)	31 (13,260/43357)

See footnote to Table 1 for description of containers

In a few cases, field workers did not record if the container was covered or not

Table 3 shows the mean entomological indices by region. There was generally little variation, except that the Breteau index was lower in Costa Grande, and the pupa per person index was lower in Acapulco. This latter probably reflects the higher population density in Acapulco.

**Table 3:** Entomological indices in the three regions

Index	Mean index% (proportion); 95% CI			
	Acapulco	Costa Grande	Costa Chica	Total
Household index	13.5 (551/4094); 12.4–14.5	14.2 (559/3946); 13.1–15.3	14.8 (566/3816); 13.7–16.0	14.1 (1676/11,856); 13.5–14.8
Container index	6.3 (1098/17,494); 5.9–6.6	6.5 (862/13,349); 6.0–6.9	6.9 (998/14,510); 6.5–7.3	6.5 (2958/45,353); 6.3–6.8
Breteau index	27 (1098/4094); 25.5–28.2	21.8 (862/3946); 20.6–23.2	26.2 (998/3816); 24.8–27.7	25 (2958/11,856); 24.9–25.7
Pupa/person index	0.08 (1536/18,372); 0.079–0.087	0.19 (3236/16,950); 0.185–0.197	0.20 (3540/18,129); 0.19–0.201	0.16 (8312/53,541); 0.152-0.168

Barrels and concrete tanks together accounted for 74% (6028/8132) of the total pupal productivity (Table 4). They are commonly used for water storage and together make up 57% (26,073/45,353) of the total number of containers (See Table 1). This contrasts with plastic tanks and water bottles, which together make up 11% of the total number of containers (see Table 1) but together account for only 1.4% (118/8132) of pupal productivity (Table 4). This is probably related to the fact that most of the plastic tanks and water bottles were covered (closed) (Table 4).

**Table 4:** Pupal productivity and other features of different water containers

Container type	N	Inside the house (%)	Containing temephos (<2 months old)(%)	Covered(%)	Used for drinking and cooking water (%)	Container index (%)	Pupal productivity
Concrete tanks	9358	19.0	40.4	8.2	1.8	8.0	*N* = 4216 mean = 0.451 SD = 5.263 50.7%
Barrels/drums	16,717	23.6	17.7	35.0	12.2	4.4	*N* = 1902 mean = 0.114 SD = 1.987 22.9%
Plastic tanks	3369	5.1	33.3	85	4.0	1.9	*N* = 107 mean = 0.032 SD = 0.536 1.3%
Plastic bottles	1557	68.9	1.2	81.4	70.5	0.7	*N* = 11 mean = 0.007 SD = 0.154 0.1%
Buckets	8729	27.0	5.1	25	10.0	2.5	*N* = 472 mean = 0.054 SD = 1.181 5.6%
Other water storage containers	4601	18.2	7.0	9.3	9.9	4.6	*N* = 882 mean = 0.19 SD = 2.52 10.6%
Discarded articles	618	9.0	24.4	N/A	N/A	9.8	*N* = 171 mean = 0.277 SD = 2.94 2.0%
Plant- and flowerpots	204	38.4	5.1	N/A	N/A	14.0	*N* = 88 mean = 0.431 SD = 2.507 1.06%
Tyres	213	32.3	2.1	N/A	N/A	7.2	*N* = 463 mean = 2.17 SD = 14.14 5.6%
All containers	45,366	23.0	19.5			4.7	*N* = 8312 mean = 0.183 SD = 3.001

Pupa productivity: *N* = total number of pupas for all containers of that type; mean = mean number of pupae per container; SD = standard deviation of mean; percentage = (total number of pupae from that container type/overall total number of pupae) × 100

### Temephos, covering containers and presence of larvae and pupae

Larvae and/or pupae were found in 5.8% (336/5747) of containers with active temephos (applied within the last 2 months), and in 1.8% (189/10,562) of containers which were covered at the time of the survey. In an analysis taking into account the effect of temephos and the effect of container coverage, the two factors were independently associated with a reduced likelihood of pupae or larvae in the container, but the effect of container coverage (OR 0.20, 95% CIca 0.15–0.27) was stronger than the effect of temephos (OR 0.66, 95% CIca 0.53–0.84).

Table 5 shows the results of the bivariate analysis of associations with the household index. A household was more likely to have at least one container positive for *Ae. aegypti* larvae or pupae if: there were more than four water containers in the household; the household used anti-mosquito products; the household head did not have remunerated employment; and the household head had less than 6 years of primary education. Households with temephos in at least one water container were less likely to be positive for larvae or pupae.

**Table 5:** Bivariate associations with household index

Variable	Positive households^a^		OR	95% CIca
	Proportion	**%**		
Number of water containers in household				
4–51	756/4646	16.3	**1.55**	**1.33–1.80**
1–3	820/7349	11.2		
Respondent knew about the dengue vector				
Yes	322/2302	14	1.10	0.93–1.29
No	1219/9420	12.9		
Household use				
Home	1490/11355	13.1	0.97	0.74–1.27
Business/home-business	83/618	13.4		
Area of residence				
Rural	907/6494	14	1.17	0.82–1.67
Urban	669/5501	12.2		
Household type				
Permanent or semi-permanent	942/6721	14	1.19	0.97–1.47
Temporary or provisional	627/5215	12		
Temephos in at least one water container				
Yes	468/4644	10.1	**0.63**	**0.57–0.94**
No	1108/7351	15.1		
Household uses anti-mosquito products				
Yes	745/5260	14.2	**1.17**	**1.04–1.33**
No	824/6683	12.3		
Household head with remunerated employment				
No	342/2166	15.8	**1.30**	**1.13–1.50**
Yes	1221/9704	12.6		
Region				
Acapulco	523/4131	12.7	0.94	0.64–1.37
Costa Grande &Costa Chica	1053/7864	13.4		
Education of household head				
0–5 years of primary school	748/4910	15.2	**1.35**	**1.17–1.56**
6 or more years of primary school	822/6988	11.8		
Language spoken at home				
Spanish	1457/10905	13.4	1.26	0.84–1.84
Indigenous language	117/1076	10.9		
Regular water service				
Yes	1295/10095	12.8	0.85	0.53–1.36
No	281/1076	14.8		
Garbage collection service				
Yes	932/7272	12.8	0.93	0.65–1.33
No	644/4723	13.6		

^a^A positive household had at least one container in which *Ae*. *aegypti* larvae or pupae were found

95% CIca = cluster adjusted 95% confidence interval

Bold font indicates an association significant at the 5% level

In the final model of the multivariate analysis of associations with household index in rural areas (Table 6), the factors remaining associated with the household being positive for larvae or pupae were: having more than four water containers in the household; household use of anti-mosquito products; a household head with less than 6 years of primary education; a household head without remunerated employment; and location in the Acapulco region.

**Table 6:** Multivariate model of factors associated with household index in rural areas; *N* = 6362; Clustered by site, *n* = 51

Variable	Crude OR	Adjusted OR	95%CIca
More than four water containers	1.46	1.42	1.17–1.72
Use of anti-mosquito products	1.28	1.27	1.09–1.47
Household head <6 years education	1.23	1.27	1.11–1.46
Household head without paid employment	1.38	1.31	1.08–1.59
Household in Acapulco region	1.85	1.91	1.06–3.44

OR = Odds Ratio; 95%CIca = cluster adjusted 95% confidence interval

In the final multivariate model in urban areas (Table 7), households were more likely to be positive if they had more than four water containers and if the household head had less than 6 years of education. They were less likely to be positive if they had active temephos in at least one water container.

**Table 7:** Multivariate model of factors associated with household index in urban areas; *N* = 5440; clustered by site, *n* = 39

Variable	Crude OR	Adjusted OR	95% CIca
More than four water containers	1.68	1.81	1.47–2.25
Household head <6 years education	1.44	1.39	1.17–1.66
Temephos in at least one container	0.48	0.44	0.32–0.60

OR = Odds Ratio; 95%CIca = cluster adjusted 95% confidence interval

## Discussion

The main value of the results of the study described here is that they were a key part of the evidence base for discussions about dengue prevention in the Camino Verde trial [13, 15] intervention communities. The aim of these discussions was to co-design, with communities, communication and dissemination strategies about vector control that would allow the communities to make decisions to carry out actions to prevent the reproduction of *Ae. aegypti* [22].

### Main household Ae. Aegypti breeding sites

The study provided evidence about the main mosquito breeding sites in the three coastal regions of Guerrero. This was very useful information to discuss with households. Barrels were the most common type of water container in the households, followed by concrete tanks and buckets (see Table 1). Concrete tanks alone accounted for half the total number of pupae in the households, with barrels accounting for another quarter (see Table 4). This result is partially explained by the relatively high numbers of these containers, but concrete tanks also had a high mean number of pupae per container. Households therefore need to pay particular attention to these containers. Our results about the main household breeding sites for *Ae. aegypti* are similar to those of other authors. A study in an Argentinian city reported water barrels and tanks/tubs were often infested [23] and a study in Thailand [24] found that a third of cement water tanks were infested.

### Actions to reduce Ae aegypti breeding

This study provides evidence of the importance of the simple action of keeping water containers properly covered. Taking into account the effect of temephos, covered water containers were about five times less likely to be positive for larvae or pupae, compared with open containers (OR 0.22; 95% CIca 0.15–0.27). The study findings also confirmed an effect of temephos in water containers: taking into account whether containers were covered, those with active temephos in them (less than 2 months old) were less likely to be positive for larvae or pupae (OR 0.66; 95% CIca 0.53–0.84). The effect of covering containers is clearly stronger than the effect of temephos. Phuanukoonnon et al. in northeast Thailand also found that covering drinking water jars was associated with less infestation, and showed that temephos was effective only in certain types of container in urban sites [24]. Garelli et al. in Argentina noted a relatively short and variable residual effect of temephos, with high water turnover (for example from refilling tanks from an overnight water supply) reducing the period during which temephos was active [25]. One reason for the greater effectiveness of covering containers may be the growing resistance of the dengue virus to the chemical [26].

At the time of the survey reported here, neither control measure (covering water storage containers and inserting temephos) was universally present in the households. Only plastic water tanks and water bottles had a high rate of coverage (85% and 81%), with a quarter of buckets, a third of barrels and only one in ten concrete tanks covered (Table 2). Active temephos (that is, less than 2 months old) was present in only 19% of containers, and in particular it was present in only 40% of concrete tanks and 18% of barrels, which together account for three-quarters of pupae in the households (Table 4). Increasing coverage of the government temephos programme would pose challenges, but households can easily take action themselves to cover water containers. The *brigadistas* (community mobilisers) of the Camino Verde intervention encouraged households to become involved in this sort of activity [22].

### Associations with household index

In our study, in both rural and urban sites, households with more water containers were more likely to be positive for larvae or pupae. This is not surprising, as more containers will provide more potential breeding sites for *Ae. aegypti*. The number of containers per household is a relatively crude measure, since it does not take into account the variation in container type, with some types being much more productive of pupae than others. Still, having fewer water containers is one way to minimize mosquito proliferation.

Also in all sites, households were more likely to be positive for larvae or pupae if the household head had less than 6 years of education. In rural sites, households were more likely to be positive if they were poorer, as indicated by the household head not having any paid employment. A study in Cuba found lack of employment was associated with Ae. aegypti infestation [27] while one in Colombia reported associations between *Ae. aegypti* larvae and pupae and low socio-economic indicators, including poverty and low education [11]. A study Southern Mexico [12] linked low education of the household head with the presence of high-risk containers for *Ae. aegypti* breeding.

In the present study, we did not find a significant association between area of residence (urban or rural) and household positivity for larvae or pupae. Authors from Brazil and Argentina have reported an association between poorly developed urban locations and conditions favouring breeding of *Ae. aegypti* [6, 7]. We did find that associations between other variables and household positivity were different between urban and rural sites, suggesting that some factors may operate differently in urban locations. In rural sites only, we found that households in the Acapulco region were more likely to be positive for larvae or pupae. The reasons for this are not clear, but it may be related to the relative water shortage in this region, leading to different water storage practices not fully reflected in the present analysis.

In urban sites only, households with temephos in at least one container were less likely to be positive for larvae and pupae. This may reflect a more thorough application of temephos in urban sites. On the other hand, in rural sites, households reporting the use of anti-mosquito products (such as sprays and coils) were more likely to be positive for larvae or pupae. In a cross-sectional study, we cannot be sure of the direction of associations, and it could be that those households with infestation were more bothered by adult mosquitoes and hence resorted to using anti-mosquito products. The finding certainly does not suggest that these products had any useful effect on reducing *Ae. aegypti* breeding.

### Limitations of the study

As with any cross-sectional study, we cannot draw conclusions about causality from this study, and there may be other confounders we have not been able to take into account. We cannot be sure of the direction of associations identified.

## Conclusions

This study produced findings that were useful in planning and implementing with communities the evidence-based interventions of the Camino Verde trial. It indicated that the bulk of pupal productivity in households is concentrated in the larger traditional water storage containers of concrete tanks (*pilas*) and barrels (*tambos*). It pointed to the value of covering water containers to prevent *Ae. aegypti* breeding, and the stronger effect of this compared with the effect of temephos.

## Abbreviations

Ae. aegypti: *Aedes aegypti*
CIET: *Centro de Investigación de Enfermedades Tropicales*
OR: Odds ratio
95% CIca: Cluster adjusted 95% confidence interval

## References

[1] Dujardin JC, Herrera S, Do Rosario V, Arevalo J, Boelaert M, Carrasco HJ (2010). Research priorities for neglected infectious diseases in Latin America and the Caribbean region. PLoS Negl Trop Dis.

[2] Bhatt S, Gething PW, Brady OJ, Messina JP, Farlow AW, Moyes CL (2013). The global distribution and burden of dengue. Nature.

[3] Organización Panamericana de la Salud/Organización Mundial de la Salud (2010). Dengue guías para el diagnóstico, tratamiento, prevención y control.

[4] Orgaización Panamericana de la Salud (1998). Plan Continental de ampliación e intensificación del combate a *Aedes aegypti*. Rev Panam Salud Publica/Pan Am J Public Health.

[5] Gubler DJ. *Aedes aegypti* and *Aedes aegypti*-borne disease control in the 1990s: top down or bottom up. Am J Trop Med Hyg. 1989;40:571–8. 10.4269/ajtmh.1989.40.571 2472746

[6] Maciel de Freitas R, Marques WA, Peres RC, Cunha SP, de Oliveira RL (2007). Variation in *Aedes aegypti* (Diptera:Culicidae) container productivity in a slum and a suburban district of Rio de Janeiro during dry and wet seasons. Mem Inst Oswaldo Cruz.

[7] Rubio A, Cardo MV, Vezzani D (2011). Tire-breeding mosquitoes of public health importance along an urbanisation gradient in Buenos Aires, Argentina. Mem Inst Oswaldo Cruz.

[8] Focks DA, Alexander N (2007). Multicountry study of *Aedes aegypti* pupal productivity survey methodology: findings and recommendations. Dengue Bulletin WHO.

[9] Morrison AC, Gray K, Getis A, Astete H, Sihuincha M, Foks D (2004). Temporal and geographic patterns of *Aedes aegypti* (Diptera: Culicidae) production in Iquitos, Peru. J Med Entomol.

[10] Villegas Trejo A, Che Mendoza A, González Fernández M, Guillermo May G, González Bejarano H, Dzul-Manzanilla F (2011). Control enfocado de *Aedes aegypti* en localidades de alto riesgo de transmisión de dengue en Morelos, México. Salud Pública Mex.

[11] Quintero J, Carrasquilla G, Suárez R, González C, Olano VA (2009). An ecosystemic approach to evaluating ecological, socioeconomic and group dynamics affecting the prevalence of *Aedes aegypti* in two Colombian towns. Cad Saúde Pública.

[12] Danis-Lozano R, Rodríguez MH, Hernández-Avila M (2002). Gender-related family head schooling and *Aedes aegypti* larval breeding risk in southern Mexico. Salud Publica Mex.

[13] Andersson N, Nava-Aguilera E, Arostegui J, Morales-Perez A, Suazo-Laguna H, Legorreta-Soberanis J (2015). Camino Verde (Green way) to dengue prevention: a pragmatic cluster-randomized controlled trial of evidence-based community mobilization in Nicaragua and Mexico. BMJ.

[14] Instituto Nacional de Estadística y Geografía. http://www.inegi.org.mx/est/contenidos/espanol/sistemas/aee12/estatal/GRO/default.htm. Accessed 1 May 2017.

[15] Andersson N, Arostegui J, Nava-Aguilera E, Harris E, Ledogar RJ. Camino Verde (Green Way): evidence-based community mobilisation for dengue control in Nicaragua and Mexico: feasibility study and study protocol for a randomised controlled trial. BMC Public Health. 2017;17(Suppl 1): doi:10.1186/s12889-017-4289-5. 10.1186/s12889-017-4289-5 PMC5506595 28699570

[16] Lauritsen JM, Bruus M. EpiData (version 3). A comprehensive tool for validated entry and documentation of data. Odense: The EpiData Association; 2003–2008.

[17] Andersson N, Mitchell S (2002). CIETmap: free GIS and epidemiology software from the CIETgroup, helping to build the community voice into planning.

[18] Andersson N, Mitchell S (2006). Epidemiological geomatics in evaluation of mine risk education in Afghanistan. Int J Health Geogr.

[19] Mantel N, Haenszel W (1959). Statistical aspects of the analysis of data retrospective studies of disease. J Natl Cancer Inst.

[20] Andersson N, Lamothe G (2011). Clustering and meso-level variables in cross-sectional surveys: an example of food aid during the Bosnian crisis. BMC Health Serv Res.

[21] Woolf B (1955). On estimating the relation between blood group and disease. Ann Hum Genet.

[22] Morales-Pérez A, Nava-Aguilera E, Legorreta-Soberanis J, Paredes-Solís S, Balanzar-Martínez A, Serrano-de los Santos FR, et al. Which Green Way: description of the intervention for mobilising against Aedes aegypti under difficult security conditions in southern Mexico. BMC Public Health. 2017;17(Suppl 1): doi:10.1186/s12889-017-4300-1. 10.1186/s12889-017-4300-1 PMC5506570 28699562

[23] Costa F, Fattore G, Abril M (2012). Diversity of containers and buildings infested with *Aedes aegypti* in Puerto Iguazú, Argentina. Cad Saúde Pública.

[24] Phuanukoonnon S, Mueller I, Bryan JH (2005). Effectiveness of dengue control practices in household water containers in Northeast Thailand. Tropical Med Int Health.

[25] Garelli FM, Espinosa MO, Weinberg D, Trinelli MA, Gürtler RE (2011). Water use practices limit the effectiveness of a temephos-based *Aedes aegypti* larval control program in northern Argentina. PLoS Negl Trop Dis.

[26] Rodriguez MM, Bisset JA, Fernandez D (2007). Levels of insecticide resistance and resistance mechanisms in *Aedes aegypti* from some Latin American countries. J Am Mosq Control Assoc.

[27] Spiegel JM, Bonet M, Ibarra AM, Pagliccia N, Ouellette V, Yassi A (2007). Social and environmental determinants of *Aedes aegypti* infestation in Central Havana: results of a case–control study nested in an integrated dengue surveillance programme in Cuba. Tropical Med Int Health.

